# Prevalence of dizziness in patients with temporomandibular disorders: a systematic review and meta-analysis

**DOI:** 10.22514/jofph.2026.044

**Published:** 2026-07-12

**Authors:** Rayan Ibrahim H Binduhayyim, Sunil Kumar Vaddamanu, Masroor Ahmed Kanji, Rajesh Vyas, Marco Di Blasio, Gabriele Cervino, Maria Maddalena Marrapodi, Giuseppe Minervini

**Affiliations:** ^1^Department of Allied Dental Health Sciences, College of Applied Medical Science, King Khalid University, 61421 Abha, Saudi Arabia; ^2^Department of Biomedical, Surgical and Dental Sciences, University of Milan, 20122 Milan, Italy; ^3^Morphological and Functional Images, Department of Biomedical and Dental Sciences, G. Martino Polyclinic, University of Messina, 98125 Messina, Italy; ^4^Saveetha Dental College and Hospitals, Saveetha Institute of Medical and Technical Sciences (SIMATS), Saveetha University, 600077 Chennai, India; ^5^Department of Woman, Child and General and Specialized Surgery, University of Campania “Luigi Vanvitelli”, 80138 Naples, Italy; ^6^Multidisciplinary Department of Medical-Surgical and Dental Specialities, University of Campania “Luigi Vanvitelli”, 80138 Naples, Italy

**Keywords:** Temporomandibular disorders, Dizziness, Vertigo, Otological symptoms

## Abstract

**Background**: Temporomandibular disorders (TMDs) are characterised by pain and dysfunction in the temporomandibular joints and surrounding muscles. Dizziness is a frequent symptom reported by TMD patients, but the prevalence of dizziness in this population remains unclear. **Methods**: A systematic review and meta-analysis was conducted to identify relevant studies assessing the prevalence of dizziness in TMD patients. Seven electronic databases were searched using a combination of Boolean operators and Medical Subject Headings (MeSH) keywords to maximise sensitivity and specificity. **Results**: The meta-analysis revealed a significant association between TMDs and dizziness in descriptive studies (pooled odds ratio (OR): 0.42, 95% confidence interval (CI): 0.20–0.88) but found a non-significant association in analytical studies (pooled OR: 0.55, 95% CI: 0.19–1.59). Heterogeneity was low in analytical studies (0%), while descriptive studies showed higher heterogeneity (96%). The studies included in the review generally reported a significant prevalence of vertigo and other aural symptoms in TMD patients. Some investigations highlighted the value of sociodemographic characteristics and the statistical association between TMD and vestibular symptoms. Furthermore, dizziness was quantitatively attributed to TMDs in some studies, while others reported a correlation between TMDs and otolaryngological symptoms. **Conclusions**: The current review demonstrated that TMDs are associated with increased dizziness and vestibular symptoms, particularly in descriptive research. A causal relationship could not be established due to the heterogeneity and observational nature of the studies included. These findings emphasise the need for careful interpretation and recommend prospective studies to clarify the relationship. When diagnosing and treating TMD patients, clinicians should consider these symptoms together. **The PROSPERO Registration**: CRD420251026371.

## 1. Introduction

Temporomandibular disorders (TMDs) refer to a diverse range of musculoskeletal and neuromuscular problems affecting the temporomandibular joint (TMJ) and its surrounding structures [1, 2]. They have a significant impact on overall quality of life and are characterised by symptoms such as pain, joint noises, restricted movement of the jaw, and pain in the head and face. TMDs are prevalent in approximately 10–15% of the global population, with a greater predilection for women [3, 4, 5].

In the area of vestibular disorders, early in the last century, the Barany Society [6] proposed an empirical differentiation between dizziness and vertigo. Dizziness is further divided into vertigo and non-vertigo categories. Different sets of symptoms are observed with vertigo and non-vertigo. In contrast, the American Otorhinolaryngologic society [7, 8, 9] has a broader definition, arguing that dizziness is a broader term that includes vertigo as a subcategory. A clear example of dizziness prevalence is walking-related dizziness, which affects a great number of people (estimates indicate that 15%–35% of people experience dizziness at least once in their lifetime). This vestibular malfunction might cause severe consequences, such as inability to work, disruption of normal life, and reduced quality of life [10, 11]. Another relevant condition is multicanal benign paroxysmal positional vertigo (mc-BPPV), a vestibular disorder that may be mistaken for dizziness in relation to TMD.

TMDs result from a multifactorial combination of biomechanical, neuromuscular, and psychosocial factors [12]. This combination of factors can lead to the development of a variety of comorbid conditions, such as orofacial pain, headache, and sleep disturbances [13]. Dizziness—whether feeling light-headed, off balance, or even vertigo—is a frequently ignored, yet potentially disabling symptom of TMDs. The exact pathophysiological mechanisms that cause dizziness are still unclear in patients with TMD. However, changes in how the TMJ processes information about body position and pain, along with heightened sensitivity in the central nervous system and emotional factors, are believed to play a role in the development of this symptom [13, 14, 15, 16, 17].

Another area that should be explored is central sensitisation in TMD patients. Central sensitisation refers to heightened neuronal signalling within the central nervous system that leads to increased pain perception and exaggerated responses to sensory stimuli. This mechanism is increasingly prevalent in chronic pain conditions and can be central to explaining why patients with TMD experience not only localised symptoms but also more generalised sensory deficits like dizziness and loss of balance. The use of a neurophysiological framework clarifies the various mechanisms of TMD-related dizziness.

Dizziness related to TMD may have a significant impact on functioning, social life, and overall quality of life [11, 18, 19, 20]. Understanding this complex phenomenon is very relevant for our overall understanding—from the various characteristics of TMDs to the extreme heterogeneity of associated symptoms. Therefore, the purpose of this systematic review and meta-analysis is to synthesize and consolidate all the data related to the prevalence of dizziness in TMD patients in order to better understand the magnitude of the problem and guide the development of personalized treatment approaches.

## 2. Materials and methods

### 2.1 Eligibility criteria

The present systematic review performed according to Preferred Reporting Items for Systematic Reviews and Meta-Analyses (PRISMA) guidelines (**Supplementary material**) [21] to maintain the transparency, reproducibility and rigour of methodological quality and was registered with the International Prospective Register of Systematic Reviews (PROSPERO) (CRD420251026371). In order to accomplish our aim, the Population, Exposure, Comparator, Outcome (PECO) framework was used, which enabled us to formulate the research question and identify relevant studies. In our instance, the PECO framework was defined as follows: Population (P): people of all ages and sexes; Exposure (E): TMDs; Comparator (C): not applicable; and Outcome (O): prevalence of dizziness in patients with TMDs. The inclusion and exclusion criteria used in this review are detailed in Table 1.

**Table 1.** Selection criteria devised for the review.

Criteria	Inclusion	Exclusion
Study Design	Observational studies (cross-sectional, case-control, cohort)	Interventional studies, reviews, case reports, opinion articles
Study Population	Patients with TMDs	Patients with other orofacial pain conditions, healthy controls
Exposure	Presence of TMDs	Absence of TMDs, other medical conditions
Outcome	Prevalence of dizziness	Other symptoms or outcomes (*e.g.*, pain, quality of life)
Language	No limitation	
Publication date	No limitation	

*TMDs: temporomandibular disorders.*

### 2.2 Search strategy

The search covered electronic databases PubMed, Scopus, Web of Science, Embase, PsycINFO, CINAHL, and the Cochrane Library for eligible studies. To improve the sensitivity and specificity of the search a combination of Boolean operators and MeSH keywords were applied in the database. Studies published from the start to the present period without any restrictions on language were included. The results of the search are presented in Table 2.

**Table 2.** Search strings/phrases utilized across the databases.

Database	Search string
PubMed	((“temporomandibular joint disorders” OR “temporomandibular joint pain” OR “craniofacial pain”) AND (“dizziness” OR “vertigo” OR “lightheadedness”)) AND (“prevalence” OR “incidence” OR “epidemiology”)
Scopus	(TITLE-ABS-KEY (“temporomandibular joint” OR “TMJ” OR “craniofacial pain”) AND TITLE-ABS-KEY (“dizziness” OR “vertigo” OR “lightheadedness”)) AND TITLE-ABS-KEY (“prevalence” OR “incidence” OR “epidemiology”)
Web of Science	(TS = (“temporomandibular joint disorders” OR “temporomandibular joint pain” OR “craniofacial pain”) AND TS = (“dizziness” OR “vertigo” OR “lightheadedness”)) AND TS = (“prevalence” OR “incidence” OR “epidemiology”)
Embase	(“temporomandibular joint disorder”/exp OR “temporomandibular joint pain”/exp OR “craniofacial pain”/exp) AND (“dizziness”/exp OR “vertigo”/exp OR “lightheadedness”/exp) AND (“prevalence”/exp OR “incidence”/exp OR “epidemiology”/exp)
PsycINFO	(DE (“temporomandibular joint disorders” OR “temporomandibular joint pain” OR “craniofacial pain”)) AND (DE (“dizziness” OR “vertigo” OR “lightheadedness”)) AND (DE (“prevalence” OR “incidence” OR “epidemiology”))
CINAHL	(MH “Temporomandibular Joint Disorders” OR MH “Temporomandibular Joint Pain” OR MH “Craniofacial Pain”) AND (MH “Dizziness” OR MH “Vertigo” OR MH “Lightheadedness”) AND (MH “Prevalence” OR MH “Incidence” OR MH “Epidemiology”)
Cochrane Library	(temporomandibular joint disorders OR temporomandibular joint pain OR craniofacial pain) AND (dizziness OR vertigo OR lightheadedness) AND (prevalence OR incidence OR epidemiology)

*TS: Topic Search; DE: Descriptor; MH: Medical Heading.*

A standardized data extraction protocol was adopted to facilitate a thorough and systematic data extraction process from the studies included. This protocol outlined the specific information to be retrieved, the types of data extraction, the means through which conflicts would be resolved, and the procedures for each type of data retrieved. Using a standard data extraction template, two reviewers independently extracted data from each study. These data items are described below:

The form was pilot-tested on a selected sample of studies to ensure consistency and specificity. Data items for the review were identified based on their relevance to the research topic, and their potential contribution to the synthesis of results. Data were extracted on study characteristics for each study including author, year, country, study design, sample size, and duration, participant demographics (age, gender, and TMD diagnosis), exposure (presence of TMDs), outcome (prevalence of dizziness), and quality of study (risk of bias, potential confounding variables, and statistical methods). Data on the definition and measurement of dizziness, and the diagnostic criteria for the classification of TMDs, were extracted.

### 2.3 Assessment of bias

For the assessment of risk of bias in the included studies [22], the Appraisal tool for Cross-sectional (X-sectional) Studies (AXIS) and Risk Of Bias In Non-randomized Studies of Interventions (ROBINS-I) (clinical studies) tool [23, 24] were used, respectively.

### 2.4 Statistical analysis

The random-effects (RE) model was used to carry out the meta-analysis, accounting for expected heterogeneity between studies. The main outcome was the prevalence of dizziness in TMD patients, expressed as an odds ratio (OR) with a 95% confidence interval (CI). Forest plots were then used to display the results and display the OR and 95% CI for each study, plus pooled estimates and corresponding 95% CI data. Review Manager (RevMan) Version 5.4 (The Cochrane Collaboration, Copenhagen, Denmark), a software developed by the Cochrane Collaboration for meta-analysis and systematic review. The *I*^2^ statistic was used to quantify the proportion of variation in the effect estimates attributable to heterogeneity between studies. Multiple database searches minimized the risk of missing unpublished, or selectively reported literature.

## 3. Results

### 3.1 Study selection process

Initially, 311 records were identified from databases, while no records were obtained from registers. After removing duplicates (n = 47), 264 records were screened, and 28 were excluded due to full-text unavailability. Of the remaining 236 reports sought for retrieval, 19 were not retrieved, leaving 217 reports assessed for eligibility. A total of 210 reports were excluded due to various reasons, including not meeting the PECO criteria (n = 51), being off-topic (n = 48), being literature reviews (n = 29), scoping reviews (n = 40), or grey literature (n = 42). Ultimately, 7 studies [7, 25, 26, 27, 28, 29, 30] were included in the review, as illustrated in Fig. 1.

**Fig. 1.** Schematic of the study inclusion process for the review. PECO: Population, Exposure, Comparator, Outcome.

### 3.2 Domains of bias observed

Using the AXIS tool, Akhter *et al*. [25] and Song *et al*. [28] demonstrated moderate overall bias, mostly due to selection bias. In contrast, Guimarães *et al*. [26] showed low overall bias, with minimal concerns across all domains. Aldè *et al*. [7] showed an overall low bias, but moderate concerns in some domains. Honorato *et al*. [27] and Tuz *et al*. [30] showed moderate overall bias, mainly resulting from selection bias and detection bias (Fig. 2, Ref. [25, 26, 28]; Fig. 3, Ref. [7, 27, 29, 30]).

**Fig. 2.** Bias assessment using the AXIS tool.

**Fig. 3.** Bias assessment using the ROBINS-I tool.

### 3.3 Demographic variables assessed

Table 3 (Ref. [7, 25, 26, 27, 28, 29, 30]) shows the demographic characteristics of the included studies. The studies were conducted in various countries, including Japan [25], Italy [7], Brazil [26, 27], South Korea [28], Sweden [29], and Turkey [30], indicating a geographically diverse sample. The study protocols included were primarily descriptive [25, 26, 28], with one retrospective study [7], two case-control studies [27, 29], and one prospective study [30]. The sample sizes ranged from 60 [27] to 2059 [28], with a total of 5393 participants across all studies. The mean age of the participants varied across studies, ranging from 18.6 ± 2.1 years [25] to 47 ± 13 years [29]. The age distribution was mainly skewed toward younger individuals, with six studies having a mean age below 40 years [7, 25, 26, 27, 28, 30]. The male-to-female ratio varied across studies. A near-balanced distribution was observed in two studies [25, 29], while the remaining studies showed a female-predominant sample [7, 26, 27, 28, 30]. None of the included studies reported a male-predominant population.

**Table 3.** Demographic characteristics of the included papers.

Study ID	Year	Country	Protocol	Sample size (n)	Mean age (in year)	Male:Female ratio
Akhter *et al*. [25]	2013	Japan	Cross-sectional	1930	18.6 ± 2.1	981:949
Aldè *et al*. [7]	2022	Italy	Retrospective	400	39.6 ± 15.6	99:301
Guimarães *et al*. [26]	2023	Brazil	Cross-sectional	623	28.6 ± 9.6	239:384
Honorato *et al*. [27]	2022	Brazil	Observational case-control	60	36.3 ± 12.3	16:44
Song *et al*. [28]	2018	South Korea	Cross-sectional	2059	38.6 ± 0.42	750:1309
Tullberg *et al*. [29]	2006	Sweden	Observational case-control	121	47 ± 13	64:56
Tuz *et al*. [30]	2003	Turkey	Prospective	200	29.6	35:165

### 3.4 Groups and parameters observed

Table 4 (Ref. [7, 25, 26, 27, 28, 29, 30]) shows the examination of the relationship between TMDs and dizziness across the included studies. Each study employed different methodologies and participant groups to explore this correlation. Akhter *et al*. [25] compared TMD-negative and TMD-positive groups, focusing on aural symptoms such as tinnitus, otalgia, and vertigo, as well as headache, depression, and shoulder pain.

**Table 4.** Correlation between dizziness and TMD as observed across the included studies.

Study name	Groups observed	Parameters observed	Dizziness/vertigo levels observed	Statistical observations	Overall conclusion
Akhter *et al*. [25]	TMD-negative and TMD-positive groups	Aural symptoms (tinnitus, otalgia, vertigo), headache, depression, and shoulder pain	12.4% of TMD-negative and 38.1% of TMD-positive groups reported vertigo	Significant association between TMD and vertigo (OR 1.34, 95% CI 1.02–1.75)	TMD is associated with a higher prevalence of vertigo and other aural symptoms
Aldè *et al*. [7]	TMD patients (n = 400)	Otological symptoms (aural fullness, vertigo, tinnitus)	19.8% reported vertigo	New-onset otological symptoms reported by 76% of TMD patients, with vertigo being the third most common symptom	TMD is associated with a high prevalence of otological symptoms, including vertigo
Guimarães *et al*. [26]	523 participants with TMD	Sociodemographic questionnaire, DHI, FAQ, DC/TMD	79.9% reported dizziness, tinnitus, or nystagmus	No significant association between VS and QoL (*p* > 0.05)	Association between VS and sociodemographic variables, including female gender, young adults, and students
Honorato *et al*. [27]	20 patients with TMD and 40 controls	DHI adapted into Portuguese	50% of TMD patients had severe dizziness (score 61–100)	Statistically significant difference in aural fullness and otalgia symptoms between groups (*p* < 0.01)	Association between TMD and dizziness, with TMD as a probable cause in 50% of cases
Song *et al*. [28]	Koreans aged ≥20 years	Ophthalmologic and otolaryngological symptoms, TMD prevalence	1.52 (95% CI, 1.27–1.82)	OR: 1.52 (95% CI, 1.27–1.82) for dizziness/balance disorder, 1.97 (95% CI, 1.70–2.27) for tinnitus	TMD prevalence is associated with ophthalmologic and otolaryngological symptoms, including dizziness and vertigo, with a statistically significant odds ratio of 1.52 for dizziness/balance disorder
Tullberg *et al*. [29]	TMD patients with tinnitus	Tinnitus, oral parafunctions, quality of life, TMJ/masticatory muscle examination	Not explicitly reported	89% of patients rated tinnitus as moderate to severe, 73% reported a reduction in tinnitus after treatment	Tinnitus is a common symptom in TMD patients and can be improved with treatment
Tuz *et al*. [30]	TMD patients (MPD, ID, MPD + ID) and the control group	Otalgia, tinnitus, vertigo, hearing loss, pure tone audiometry, impedance test, and reflex tympanometry	Vertigo was significantly higher in TMD patients than in the control group	No significant difference in prevalence of otalgia, tinnitus, vertigo, and hearing loss among TMD groups, otalgia was higher in TMD patients than in the control group	TMD patients have a higher prevalence of otalgia, tinnitus, and vertigo compared to asymptomatic individuals

*TMD: temporomandibular disorder; OR: odds ratio; CI: confidence intervals; TMJ: temporomandibular joint; DHI: Dizziness Handicap Inventory; DC: Diagnostic Criteria; MPD: myofascial pain dysfunction; ID: internal derangement; VS: vestibular symptoms; QoL: quality of life; FAQ: Fonseca Anamnestic Questionnaire.*

Aldè *et al*. [7] examined a cohort of 400 TMD patients, specifically investigating otological symptoms including aural fullness, vertigo, and tinnitus. Guimarães *et al*. [26] involved 523 participants with TMD and utilized a sociodemographic questionnaire, along with the Dizziness Handicap Inventory (DHI), Fonseca Anamnestic Questionnaire (FAQ), and Diagnostic Criteria for TMD (DC/TMD). Honorato *et al*. [27] conducted a study with 20 TMD patients and 40 control subjects, employing the Portuguese-adapted version of the DHI.

Song *et al*. [28] focused on Koreans aged 20 years and older, examining both ophthalmologic and otolaryngological symptoms in relation to TMD prevalence. Tullberg *et al*. [29] specifically targeted TMD patients with tinnitus, assessing tinnitus, oral parafunctions, quality of life, and conducting TMJ/masticatory muscle examinations. Tuz *et al*. [30] compared TMD patients categorized into myofascial pain dysfunction (MPD), internal derangement (ID), and a combination of both (MPD + ID) with a control group.

### 3.5 Dizziness-associated observations

Akhter *et al*. [25] reported that 12.4% of TMD-negative individuals and 38.1% of TMD-positive individuals experienced vertigo, indicating a higher prevalence of vertigo among those with TMD. The statistical analysis revealed a significant association between TMD and vertigo with an odds ratio (OR) of 1.34 (95% CI 1.02–1.75). Aldè *et al*. [7] found that 19.8% of their TMD patients reported vertigo. In addition, 76% of TMD patients reported new-onset otological symptoms, with vertigo ranking third among them, emphasising the high incidence of vertigo in TMD patients. Guimarães *et al*. [26] found that 79.9% of TMD patients experienced dizziness, tinnitus, or nystagmus. However, no statistically significant relationship between vestibular symptoms (VS) and quality of life (QoL) was found (*p* > 0.05), indicating that, while dizziness is common, it may not have a substantial impact on overall QoL.

Honorato *et al*. [27] reported that 50% of TMD patients had severe dizziness with scores between 61 and 100. Aural fullness and otalgia symptoms were significantly different between TMD patients and controls (*p* < 0.01), implying that dizziness and associated symptoms were notably more severe in TMD patients. Song *et al*. [28] did not report dizziness directly, but reported OR values for the association between TMD and dizziness/balance disorders (OR: 1.52, 95% CI, 1.27–1.82) as well as tinnitus (OR: 1.97, 95% CI, 1.70–2.27), showing a strong association linking TMD and vestibular symptoms.

Tullberg *et al*. [29] reported that 89% of TMD patients rated their tinnitus as moderate to severe, with 73% reporting a reduction in tinnitus after treatment, although they did not explicitly report dizziness levels. This study focused primarily on tinnitus severity and its correlation with TMD. Tuz *et al*. [30] found that vertigo was significantly higher in TMD patients compared to the control group. However, there was no significant difference in the prevalence of otalgia, tinnitus, vertigo, or hearing loss among the different TMD groups. Otalgia was noted to be higher in TMD patients compared to controls.

Overall, it was found that TMD patients experienced higher rates of dizziness and vertigo compared with those without TMD.

### 3.6 Meta-analysis observations

The forest plots presented in Fig. 4 (Ref. [29, 30]) and Fig. 5 (Ref. [25, 28]) display the ORs and 95% CIs for the frequency of dizziness associated with TMDs in analytical (non-randomized) and descriptive studies, respectively, assuming the RE model. In Fig. 4, the pooled OR for the frequency of dizziness in observational studies was 0.55 (95% CI: 0.19, 1.59), indicating no significant association between TMDs and dizziness. The *I*^2^ statistic was 0%, suggesting low heterogeneity between the two included studies [29, 30]. The test for overall effect was not significant (*p* = 0.27).

**Fig. 4.** Frequency of dizziness observed associated with TMDs in non-randomised trials. TMD: temporomandibular disorder; CI: confidence intervals; M-H: Mantel-Haenszel.

**Fig. 5.** Frequency of dizziness observed associated with TMDs in descriptive studies. TMD: temporomandibular disorder; CI: confidence intervals; M-H: Mantel-Haenszel.

The pooled OR for the prevalence of dizziness in descriptive studies was 0.42 (95% CI: 0.20, 0.88), suggesting a significant association between TMDs and dizziness and a decreased likelihood of dizziness in the TMD negative group, as seen in Fig. 5. This means that the TMD-positive group is around 2.38 times more likely (1/0.42) to experience dizziness compared with control groups.

The *I*^2^ statistic was 96%, suggesting high heterogeneity between the two included studies [25, 28]. The test for overall effect was significant (*p* = 0.02). The high heterogeneity indicates substantial variations in study sample, diagnostic criteria for TMD, assessment tools for dizziness, and methodological quality. The use of a random-effects model accounts for between-study variability; however, the significant heterogeneity limits the reliability of the pooled effect estimate and necessitates careful interpretation. The limited number of included studies precluded the performance of subgroup or sensitivity analyses to further investigate sources of heterogeneity.

## 4. Discussion

The current systematic review and meta-analysis were conducted to assess the occurrence of dizziness and vertigo in TMD patients. A definite association was noted between dizziness and TMD patients among the studies included.

Akhter *et al*. [25], Aldè *et al*. [7], and Tuz *et al*. [30] were similar in their findings of a significant prevalence of vertigo and other aural symptoms in TMD patients. Guimarães *et al*. [26] and Song *et al*. [28] similarly supported these associations, but provided additional factors and statistical techniques to quantify and explain the findings. Honorato *et al*. [27] reported a specific quantification of dizziness attributable to TMD, while Tullberg *et al*. [29] focused more closely on tinnitus as a prominent TMD symptom. Collectively, these investigations corroborated the overall inference that TMD is significantly linked with auditory symptoms, including vertigo; however, they differed in the specificity and extent of their findings. Akhter *et al*. [25] and Aldè *et al*. [7] both reported a robust relationship between TMD and vertigo, with Akhter *et al*. [25] explicitly noting a higher prevalence of vertigo and other aural symptoms in TMD-positive individuals compared to TMD-negative individuals. Aldè *et al*. [7] also described a considerable prevalence of otological symptoms, especially vertigo, in TMD patients. These observations parallel the high frequency of vertigo observed in the TMD cohort.

Guimarães *et al*. [26] concentrated on the relationship between vestibular symptoms (VS) and sociodemographic features of TMD patients, such as female sex, younger age groups, and students. This study recognized associations that differed from those reported by Akhter *et al*. [25] and Aldè *et al*. [7] by including demographic characteristics as influential variables, thereby providing a more nuanced perspective on the epidemiology of these symptoms. Honorato *et al*. [27] identified TMD as a possible cause of dizziness in 50% of cases, with this study also recognizing a link between TMD and dizziness. However, its quantitative estimation of the proportion of dizziness due to TMD was more specific than that reported in the other research.

Song *et al*. [28] found a significant association between the prevalence of TMD and ophthalmologic and/or otolaryngologic symptoms, such as dizziness and vertigo, and an odds ratio of 1.52 for dizziness/balance disorder. This study used statistical measures to test the strength of the correlation, which was similar to the approach used by Akhter *et al*. [25], though it also expanded the scope to include symptoms beyond vertigo. Tuz *et al*. [30] confirmed the presence of otalgia, tinnitus, and vertigo in TMD patients compared to asymptomatic individuals, which closely matched the results of Akhter *et al*. [25] and Aldè *et al*. [7]. However, Tuz *et al*. [30] did not find significant differences in the prevalence of these symptoms among different TMD subgroups, which adds complexity to the understanding of symptom distribution in TMD patients.

Upon comparing and reviewing the findings of our work with other reviews [31, 32, 33, 34, 35], we discovered both similarities and dissimilarities. Similarities were observed in the strong relationship between TMDs and otologic signs and symptoms, including vertigo, otalgia, tinnitus, and hearing loss. Our study, like that of Omidvar *et al*. [31], established the comorbid link between TMDs and otologic symptoms (OSs), with a significant prevalence of dizziness in TMD patients. Similarly, Bury *et al*. [35] observed a significant prevalence of otologic signs and symptoms in adult patients with TMD, with ear fullness being the most prevalent complaint. Tavares *et al*. [33] and Stechman-Neto *et al*. [34] also reported that conservative treatment approaches, including occlusal splint therapy, exercises, and counselling, may improve otologic signs and symptoms in patients with TMD.

Dissimilarities were identified in the study designs, methodology, and outcomes. Hernández-Nuño *et al*. [32] reviewed the scientific information on the pathogenesis and management of otologic problems in individuals with TMD but did not present a systematic review or meta-analysis. Tavares *et al*. [33] and Stechman-Neto *et al*. [34] focused on the effectiveness of conservative interventions on otologic signs and symptoms in patients with TMD, whereas our study assessed the prevalence of dizziness in individuals with TMD.

Recent literature has highlighted associations between alterations in spinal movements, cervicogenic dizziness, and TMDs [15]. However, the nature of the relationship between dizziness and TMD remains unclear, with ongoing debates surrounding potential causal links. Other proposed mechanism explaining the association between otoneurologic symptoms and TMD include the transfer of mechanical energy from the TMJ to the middle ear by the discomalleolar ligament, irritation of the auriculotemporal nerve, and hypertonicity of muscles innervated by the trigeminal nerve [18, 19, 20].

Several studies have suggested that otoneurological symptoms are etiologically associated with TMDs [2, 18, 20]. The anatomical closeness and embryological connection between the ear and the masseter muscle have been suggested as a possible explanation for this correlation [2, 18, 36], which can cause arterial, nerve, and ligament compression in the absence of normal TMJ positioning.

The diverse etiology of TMD [36] complicates the diagnosis of the most serious symptoms, which are not only associated with dysfunctions in the middle and inner ear but also with other functional disorders of the masticatory system [37]. Therefore, accurate diagnosis and appropriate treatment are crucial and should involve a multidisciplinary approach [38].

The higher occurrence of TMD among women in this work corroborates previous findings in the literature [39, 40]. The most prevalent symptom in both groups was tinnitus. The case group reported a greater frequency of aural fullness and otalgia, similar to the findings reported by Toledo *et al*. [12]. The complexity of embryonic ear development leads to neural connections with multiple cranial and cervical nerves and is further complicated by temporomandibular disorder-related secondary otalgia [40]. TMJ modifications represent a common cause of secondary otalgia, which is often related to trigeminal nerve pain [41].

The relationship between TMD and dizziness has been described; however, it requires further exploration, and a clearer understanding of the biological mechanisms underlying this association between TMD and dizziness. Several theories have suggested that the overlap between neurovascular and neuromuscular pathways may also explain the overlap. The connections between the temporomandibular joint, chewing muscles, and some nerves, particularly the trigeminal and vestibular systems, suggest that there is potential for an issue in one area to transfer to how we sense the next. Masticatory muscle hyperactivity or tension can generate abnormal proprioceptive signals, which may affect the perception of balance, and joint inflammation or compression from adjacent neurovascular networks (*e.g.*, auriculotemporal nerve) may indirectly affect vestibular functioning. Such interactions are important to consider before assessing clinical data, and the multidisciplinary evaluation should be key in individuals with TMD and dizziness [38].

Although our focus was on TMD as a precipitant of dizziness, we recognize that risk may be inverse when preexisting vestibular dysfunction leads to cervical muscular tension, changes in jaw function, or biomechanics of temporomandibular joints. While the exact nature of this relationship is not completely known, there could be an interaction between TMD symptoms and dizziness, or similar brain processes common to or associated with them.

The main conclusion of our meta-analysis is that cross-sectional studies presenting significant association between TMD and dizziness were inconsistent with observational (non-randomised) studies which failed to find any statistically significant association. This difference may result from different methodological aspects. Cross-sectional research tends to use larger samples with standardised questionnaires, which can detect association statistically more easily. However, these designs are vulnerable to selection bias and lack the power to assess temporal associations. Observational research, especially in smaller cohorts or having a retrospective design, may have lower statistical power and potentially have more confounding variables and greater variability in diagnostic rigour.

Furthermore, variations in participant selection, diagnostic criteria, and assessment instruments across the two study designs may have led to divergent results, highlighting the need for future research utilising standardised methodologies.

### 4.1 Limitations

The main limitation of the review is the heterogeneity in the included studies, which may have caused bias. Study designs and methodologies were different from the outcomes of the studies, which could have potentially caused inconsistent results on the prevalence of dizziness. In addition, most studies were observational and could only assess associations rather than causation between TMDs and dizziness. An element of bias could have also arisen due to the lack of standardised diagnostic criteria for both TMDs and dizziness, which further reduces the validity of the findings. The results also have limited generalisability to other populations and conditions, primarily because studies had been conducted mostly in certain geographic areas, may not necessarily be representative of other population.

Bias noted in the review may influence the results and most probably led to underestimation or overestimation of the true association between dizziness and TMD. Hence, the application of the random-effect model was considered appropriate to adjust for such variability. Nevertheless, understanding this bias only obscures the strength of the conclusion and emphasises the necessity to interpret cautiously, and highlights the importance of better-quality studies in the future.

There was variability in the diagnosis of TMD and dizziness, even though the inclusion criteria were defined using the PECO framework. Some studies utilised standard diagnostic criteria, such as DC/TMD for temporomandibular disorders, or recognised methods such as DHI to measure dizziness. Other studies relied solely on clinical examination, previous medical information, or self-reported symptoms without using formal diagnosis tools. As a result, this could have contributed to clinical and measurement heterogeneity, which would explain the lack of comparability and stability in pooled results.

The limited number of included studies, with only two studies for each meta-analysis subgroup (analytical and descriptive), also restricts this review. A small sample size reduces statistical power to detect significant associations and increase the likelihood of random error. These limitations may decrease the generalisability of results to the population at large; hence, caution should be exercised in making conclusions from the pooled estimates and clinical inference.

### 4.2 Clinical recommendations

It is necessary to promote longitudinal research and the use of consistent diagnostic criteria to advance knowledge in this field. To ensure a consistent classification of TMD cases, subsequent studies should employ internationally standardised diagnostic tools, such as DC/TMD (temporomandibular disorder), and standard equipment, such as the dizziness handicap inventory, for the standardized assessment of the severity and impact of dizziness to promote better comparability and enable more precise aggregated estimates in subsequent meta-analyses. Future studies require a long-term design to describe the development of TMD, the development of symptoms over time, and the development of dizziness or balance-related problems. Continuity should be maintained in the preparation of terminology and abbreviations in future manuscripts. Words such as “TMD”, “dizziness”, “vertigo”, and “vestibular symptoms” should be defined well and used together to eliminate ambiguity.

## 5. Conclusions

Temporomandibular disorders (TMD) may be associated with auditory symptoms such as dizziness and vertigo, according to this systematic review and meta-analysis. Numerous investigations have reported a higher prevalence of otoneurological symptoms in TMD patients, although the data should be interpreted cautiously. The variability among studies and observational approaches limit the trustworthiness of these conclusions. The reported associations should not be considered causative because the evidence does not support causality. To understand the association between TMD and dizziness, estimate prevalence, and study mechanisms, well-designed, longitudinal, interventional research using standardised diagnostic and assessment criteria are needed. Clinicians should be aware of these symptoms’ probable concurrence and use caution in diagnosis and treatment.

## Supplementary Material

Supplementary material associated with this article can be found, in the online version, at https://files.jofph.com/files/article/2057007443706036224/attachment/Supplementary%20material.docx.


